# Reviewer Thank You

**DOI:** 10.1093/sleepadvances/zpag008

**Published:** 2026-01-20

The backbone of any journal is the Editorial Advisory Board and reviewers. These individuals serve as quality control gatekeepers, volunteering their time and energy to ensure the science we publish in *SLEEP Advances* is of strong quality. We are all professionally over-extended, and so I do not take for granted the effort required to review a paper well. We are extremely grateful for the thoughtful and timely input of our Editorial Advisory Board and reviewers.

As the Editor-in-Chief, I would like to personally thank everyone who reviewed for the journal in 2025. Your time and efforts are greatly appreciated. Thank you.

Sabra Abbott

Emily Abel

Alex Agostini

Merve Aktan Süzgün

Mohd Rashid Alam

Jennifer Albrecht

Gaspare Alfì

Christelle Anaclet

Caroline Arbour

Sayaka Aritake-Okada

J. Todd Arnedt

Arturo Arrona-Palacios

Santasree Banerjee

Margaret Banker

Bengi Baran

David Barker

Mathias Basner

Celyne Bastien

Bryan Baxter

Bei Bei

Felipe Beijamini

Giulio Bernardi

Luka Biedebach

Yu Sun Bin

Dan Bing

Theresa Bjorness

Donald Bliwise

Richard Bogan

Allison Brager

Melissa Brewster

Olivia Bruce

Christian Burgess

Sarah Burkart

Anna Cai

Nicole Carmona

Patricia Carter

Nicola Cellini

Pierre Champetier

Philip Cheng

Evan Chinoy

Chin Moi Chow

Peter Colvonen

Adrien Conessa

Makayla Cordoza

Earl Crew

Stephanie Crowley

Meagan E. Crowther

Leisha Cuddihy

Tony Cunningham

Ashley Curtis

Mark Czeisler

Roberto De Luca

Dan Denis

Christopher Depner

Jaime Devine

Jessica Dietch

Derk-Jan Dijk

Cecilia Diniz Behn

Tracy Doty

Luciano Drager

Christopher Drake

Angela D'Rozario

Audrey Duarte

Royette Dubar

Brett Duce

Katherine Duggan

Jeffrey Durmer

Alexandra Ehrhardt

Greg Elder

Jason Ellis

Catherine Falla

Julia Fechner

Xueji Feng

Erin Flynn-Evans

Aaron Fobian

Peter Franzen

Flavio Frohlich

Emma Gale

Karen Gamble

Chenlu Gao

Peter Geisler

Pedro Genta

Philip Gerhman

Arijit Ghosh

Gena Glickman

Jennifer Goldschmied

Michael Grandner

Madeleine Grigg-Damberger

Diana Grigsby-Touissant

Denis Gubin

Monika Haack

Lauren Hablitz

Erin Hanlon

Brant Hasler

William Healy

Subash Heraganahally

Sarah Honaker

Kimberly Honn

Ashley Ingiosi

Francesca Ingravallo

Ryouhei Ishii

Melinda Jackson

Erica Jansen

Summer Jasinski

Shahrokh Javaheri

Matthew Jennings

Kira Vibe Jespersen

Nana Jiaod

Maria Renata José

Michal Kahn

David Kalmbach

David Kennaway

Sina Kianersi

Thomas Kilduff

Jae Kyoung Kim

Tae Kim

Kristen Knutson

Adrijana Koscec Bjelajac

Suresh Kotagal

Giri Krishnan

Daniel Kroeger

Dieter Kunz

David Kupfer

Damon Lamb

Shane Landry

Christin Lang

Olivia Larson

Esther Yuet Ying Lau

Maggie Lavender

Bastien Lechat

Clark Lee

Donald Lee

Samantha K. M. Lee

Itamar Lerner

John Lesku

Jessica Levenson

Peng Li

Zhi Li

Miranda Lim

Gosia Lipinska

Yu Liu

Zhenxing Liu

June Lo

Yiqing Lu

Jessica Lunsford-Avery

Rachel Manber

Bryce Mander

Jack Manners

Raffaele Manni

Darren Mansfield

Andjela Markovic

Nathaniel Marshall

Jennifer Martin

Luis Mascaro

Gina Mason

Nigel Mcardle

Traci McCarthy

Andrew McHill

Dana McMakin

Miguel Meira e Cruz

Maddison Mellow

Natalie Michael

Gorica Micic

Katherine Miller

Emma Milot

Hylton Molzof

Hermann L. Müller

Dimitrios Mylonas

Vincent Mysliwiec

Marie-Rachelle Narcisse

Ariel Neikrug

Xinran Niu

Sara Nowakowski

Nicolette Ognjanovski

Michele Okun

Michelle Olaithe

Ju Lynn Ong

Recep Ozdemir

Elisa Pabon

Edward Pace-Schott

Dinesh Pal

Laura Palagini

Megan Petrov

Claudia Picard-Deland

Dante Picchioni

Sara Pintwala

Gabriel Pires

Giuseppe Plazzi

Tyler Powell

Sean Prall

Federica Provini

Shaun Purcell

Mirja Quante

Shadab Rahman

Hengyi Rao

Björn Rasch

Ian Rasmussen

Niels C. Rattenborg

Abdelrahman Rayan

Anthony Reffi

Carolin Reichert

Emily Ricketts

Samantha Riedy

Rebecca Robbins

Darlynn Rojo-Wissar

Jared Saletin

Mikael Sallinen

Charli Sargent

Erin Schaeffer

Sophie Schlatter

Laura Schmidt

Sarah F. Schoch

AJ Schwichtenberg

Hannah Scott

Michael Scullin

Saman Seifpour

Akiyoshi Shimura

Tamar Shochat

Tracey Leigh Signal

Katharine Simon

Bhajan Singh

Rupsha Singh

Børge Sivertsen

Benjamin L. Smarr

A. P. Smith

Adriane Soehner

Elizaveta Solomonova

Virend Somers

Andrea Spaeth

James Spilsbury

Adam Spira

Melissa St Hilaire

Ambra Stefani

Michelle Stepan

Laura Straus

Emma Stumbles

Ilya V. Sysoev

Masako Tamaki

Takako Tanaka

Ignacio Tapia

Leila Tarokh

Stephen Thomas

Julie Tolson

Lynn Marie Trotti

Wendy Troxel

Jennifer Tudor

Orna Tzischinsky

Alexander Van Der Linden

Hans Van Dongen

Giancarlo Vanini

Jessica Vazzaz

Prem Venkatesan

Johan Verbraecken

Samuel Verges

Ram Verma

Rachel Walsh

Erin Wamsley

Bo Wang

Man Wang

Rick Wassing

Elle Wernette

Delainey Wescott

Emerson Wickwire

Ines Wilhelm

Jonathan Wisor

Patricia Wong

James Wyatt

Brendon Yee

Shawn Youngstedt

Wai Haung Yu

Phyllis Zee

Shengzi Zeng

Yizhong Zheng

Mark Zielinski

Jennifer Zitser

